# Preanalytical variables and performance of diagnostic RNA-based gene expression analysis in breast cancer

**DOI:** 10.1007/s00428-014-1652-0

**Published:** 2014-09-14

**Authors:** Christopher Poremba, Jennifer Uhlendorff, Berit M. Pfitzner, Guido Hennig, Kerstin Bohmann, Hans Bojar, Veit Krenn, Jan C. Brase, Franziska Haufe, Manuela Averdick, Manfred Dietel, Ralf Kronenwett, Carsten Denkert

**Affiliations:** 1 Institute of Pathology Munich-North, Ernst-Platz-Straße 2, 80992 Munich, Germany; 2 Center for Molecular Oncology and Molecular Pathology (ZMOMP), Hans-Günther-Sohl-Straße 12, 40235 Düsseldorf, Germany; 3 Sividon Diagnostics GmbH, Nattermannallee 1, 50829 Cologne, Germany; 4 Institute of Pathology, Charité Hospital, Campus Mitte, Charitéplatz 1, 10117 Berlin, Germany; 5 Siemens Healthcare Diagnostics Holding GmbH, Ludwig-Erhard-Straße 12, 65760 Eschborn, Germany; 6 Medical Care Center for Histology, Cytology and Molecular Diagnostics, Max-Planck-Str. 5, 54296 Trier, Germany; 7 Heinrich-Heine-University Düsseldorf, Medical Faculty, Universitätsstraße 1, 40225 Düsseldorf, Germany

**Keywords:** Breast cancer, Preanalytical, EndoPredict, Molecular pathology, Gene expression

## Abstract

Prognostic multigene expression assays have become widely available to provide additional information to standard clinical parameters and to support clinicians in treatment decisions. In this study, we analyzed the impact of variations in tissue handling on the diagnostic EndoPredict test results. EndoPredict is a quantitative reverse transcription PCR assay conducted on RNA from formalin-fixed, paraffin-embedded (FFPE) tissue that predicts the likelihood of distant recurrence in patients with ER-positive/HER2-negative breast cancer. In this study, we performed a total of 138 EndoPredict assays to study the effects of preanalytical variables such as time to fixation, fixation time, tumor cell content, and section storage time on the EndoPredict test results. A time to fixation of up to 12 h and fixation of up to 5 days did not affect the results of the gene expression test. Paired samples of FFPE sections with tumor cell content ranging from 15 to 95 % and tumor-enriched samples showed a correlation coefficient of 0.97. Test results of tissue sections that have been stored for 12 months at +4 or +20 °C showed a correlation of 0.99 when compared to results of nonstored sections. In conclusion, preanalytical tissue handling is not a critical factor for diagnostic gene expression analysis with the EndoPredict assay. The test can therefore be easily integrated into the standard workflow of molecular pathology.

## Introduction

In recent years, multigene expression assays for breast cancer prognosis have become widely available to provide additional information to standard clinical parameters and histopathological techniques supporting clinicians in treatment decisions [1–4]. RNA from formalin-fixed, paraffin-embedded (FFPE) tissues is commonly used as sample material, since fresh-frozen tissue is difficult to handle in a routine clinical care environment. Although tissue processing can be partially automated using robotic instrumentation, the current practice of sample collection, handling, and storage is not entirely standardized. This can potentially impact also gene expression assays based on RNA from FFPE tissue. Numerous studies have demonstrated that mRNA from FFPE tissues can be reliably quantified by quantitative RT-PCR [5, 6]. However, several studies already showed that preanalytical variables during tissue processing can have an influence on the quality of the RNA and thus potentially influence test results of multigene assays [7, 8]. Relevant factors include the time to fixation (TTF), the fixation time (FT), and the tissue section storage time (SST). Results from recent studies have indicated that the tumor cell content (TCC) or rather the amount of tumor-adjacent normal breast tissue can also have an effect on the performance of RNA markers [9, 10]. So far, little is known about the impact of these variables on the results of gene expression-based cancer prognosis tests, including EndoPredict.

The EndoPredict test is an RNA-based reverse transcription PCR assay that predicts the likelihood of disease recurrence in women with estrogen receptor positive (ER+) and human epidermal growth factor receptor 2 negative (HER2−) breast cancer [1]. The assay analyzes the expression level of eight cancer-related genes and four reference/control genes within the breast tumor to determine an EndoPredict score in the range of 0 to 15. Each score corresponds to a specific likelihood of breast cancer recurrence within 10 years after the initial diagnosis. Based on the calculated EP score, the patient is categorized as low (0 to <5) or high risk (5 to 15) for distant recurrence under endocrine therapy. By combining the EP score with the clinical risk factors tumor size and nodal status, a hybrid molecular and clinical risk score (EPclin) is defined. The EPclin score outperforms all conventional clinicopathological risk parameters (including Ki-67, quantitative ER, and grading [1]) and improves clinical guideline-based risk classification of patients with ER+/HER2− breast cancer [11]. EndoPredict was validated in patients from two independent phase III trials (ABCSG-6 *n* = 378, ABCSG-8 *n* = 1324) resulting in a level of evidence of I according to Simon et al. [12]. The test was analytically validated [13] and enables reliable decentral gene expression analysis in local molecular pathological laboratories [14]. The impact of variations in tissue handling, however, has not been studied so far.

Here, we performed a total of 138 EndoPredict assays to study the effects of preanalytical variations such as TTF, FT, TCC, and SST on the results of the EndoPredict breast cancer prognosis test.

## Materials and methods

### Sample material and tissue handling

**Fig. 1:**
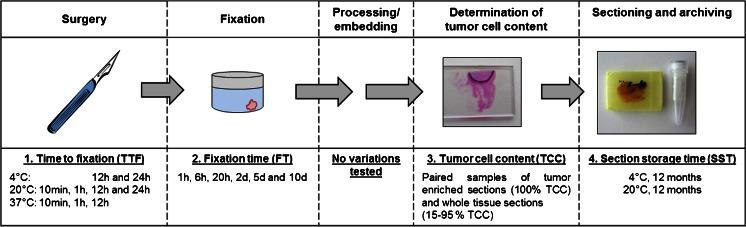
Scheme of preanaytical steps from tissue removal to archiving of the FFPE tumor material and related experiments that were performed in this study

An overview on the different preanalytical variables is shown in Fig. 1. For TTF studies, one breast tumor was dissected into nine pieces of similar size immediately after surgery. Each piece of tissue was transferred into a separate sterile Petri dish which was closed with a loose fitting lid. The remaining tumor material was used for standard histopathological diagnostics. Two tumor pieces in sterile Petri dishes were stored at 4 °C, four pieces at room temperature (20 °C), and three pieces at 37 °C without use of buffers, solutions, or stabilization reagents. After 10 min and after 1 h, respectively, one tumor piece from 20 and 37 °C was transferred from the Petri dishes into 10 % neutral buffered formalin. After 12 h, three further pieces, each from 4, 20, and 37 °C storage, were fixed in formalin. Remaining tissue pieces at 4 and 20 °C were fixed after 24 h. All specimens were fixed for ∼20 h in 10 % neutral buffered formalin (ratio fixative/tissue 20:1 or higher) followed by automated tissue processing and paraffin embedding. Two 10-μm FFPE tissue sections of each tumor piece were used for RNA isolation and analysis by EndoPredict.

In order to study the effect of the FT, another breast tumor was cut after resection into six pieces of similar size. All pieces were immediately transferred into 10 % neutral buffered formalin with a ratio of fixative to tissue of 20:1 or higher. Fixation was performed for 1 h, 6 h, 20 h, 2 days, 5 days, or 10 days. After each time point, samples were processed using an automated tissue processor (Leica, Wetzlar, Germany) followed by paraffin embedding. EP scores were determined after RNA isolation using two 10-μm tissue sections per tumor piece.

FFPE tissue samples for TCC studies were taken from 39 patients with ER+/HER2− breast cancer. The clinical data of all patients are summarized in Table 1. Five consecutive FFPE tissue sections were used for this study. One 3-μm and three 5-μm sections were mounted on glass. The 3-μm section on the glass slide was stained by H&E, and the tumor area (invasive tumor area, including interposed tumor-related stroma and ductal carcinoma in situ (DCIS)) was marked with ink by a pathologist on the glass slide. Afterward, the tumor content was assessed. Tumor content was defined as the estimated area of marked section in relation to residual tissue, whereas adipose tissue was not considered as residual tissue. In this study, the tumor content ranged from 15 to 95 %. The corresponding tumor area was copied onto the three consecutive unstained 5-μM sections on glass slides. The marked tumor areas from the three sections of the same tumor were scraped and combined into a sterile tube which contained lysis buffer for RNA isolation. These combined sections represent the tumor-enriched sample with a TCC of approximately 100 % (manually microdissected sections). In addition, a whole 10-μm section of each tumor was placed in a tube (whole section). EP and EPclin scores of whole tissue sections were compared to EP and EPclin scores of tumor-enriched, microdissected sections. EPclin scores were only calculated and compared for 38 tumor samples, since the nodal status of one patient was not available.

**Table 1:** Summary of clinical data of tissue samples used for the tumor cell content study

		Number
Tumor cell content	0–20 %	5
(whole tissue section)	21–40 %	7
	41–60 %	8
	61–80 %	9
	81–100 %	10
Tumor size	pT1b	2
	pT1c	15
	pT2	18
	pT3	4
Nodal status	positive	10
	negative	28
	unknown	1
ER status (cutoff ≥10 %)	positive	39
	negative	–
PR status (cutoff ≥10 %)	positive	31
	negative	8

Three 10-μm sections of 10 ER+/HER2− breast tumors were used to study the impact of the tissue SST on the EndoPredict test results. At the beginning of the study, RNA was isolated from a single section of each tumor, and the EP scores were determined. The remaining two tissue sections of each tumor were either stored for 12 months at 4 °C or for 12 months at 20 °C. Afterward, the RNA was isolated, and the EP scores were determined. The results were compared to EP scores of tissue sections that were analyzed at the beginning of the study (nonstored sections).

### RNA isolation from FFPE tissue

Total RNA from FFPE tissue sections was extracted by a silica-coated magnetic bead-based method using VERSANT Tissue Preparation Reagents (Siemens Healthcare Diagnostics, Tarrytown, USA) as described previously [15, 16]. Isolation was performed manually or automatically (Tissue Preparation System; Siemens Healthcare Diagnostics, Tarrytown, USA), depending on the amount of tissue sections that were analyzed within the respective study. DNA-free total RNA was eluted in 100-μl elution buffer and stored at −80 °C until use. HBB gene-specific quantitative PCR was performed to assess contamination of the eluates with residual DNA. Eluates were considered to be substantially free of DNA when Cq values above 38 were detected. In case of DNA contamination, samples were manually redigested by DNase I treatment.

### Assessment of EndoPredict score

EndoPredict tests (Sividon Diagnostics, Cologne, Germany) were performed as previously described using SuperScript III PLATINUM One-Step Quantitative RT-PCR System (Life Technologies, Darmstadt, Germany) and VERSANT kPCR Molecular System (Siemens Healthcare Diagnostics, Tarrytown, USA) [13, 14]. In brief, isolated RNA from FFPE tissue was used to assess expression levels of eight genes of interest (*AZGP1*, *BIRC5*, *DHCR7*, *IL6ST*, *MGP*, *RBBP8*, *STC2*, and *UBE2C*) and three reference genes (*CALM2*, *OAZ1*, and *RPL37A*) as well as one gene indicating DNA presence (*HBB*) by quantitative RT-PCR (qRT-PCR). PCR results were uploaded into Web-based software EndoPredict Report Generator V3.0.2 (Sividon Diagnostics, Cologne, Germany) which calculated the EP score and combined it with the clinical risk factors tumor size and nodal status to a molecular clinicopathological risk score, EPclin [1].

### Statistics

The relative expression levels of the eight genes of interest and the EP scores were calculated as described previously [1]. EPclin scores could only be determined for the TCC studies, because nodal status and tumor size of all other samples were not available.

In order to analyze the dependency of the EP score from the TTF and the FT, a reference EP score was calculated based on test results of samples with assumed standard tissue handling procedures (10 min at 20 °C prior to fixation and 20 h in formalin). Precision studies with replicate EndoPredict measurements revealed a total standard deviation of 0.25 EP score units (1.7 % of the EP score range). To account for the precision of the EndoPredict test results, a deviation of ±0.75 EP score units from the reference EP score (3-fold standard deviation of the EP score) was defined as acceptance limits for all other samples of the TTF and FT studies.

Pearson correlation coefficients (*r*) were calculated to compare EP test results of paired tissue samples with different TCC or different storage conditions.

## Results

### Effect of the time to fixation

**Fig. 2:**
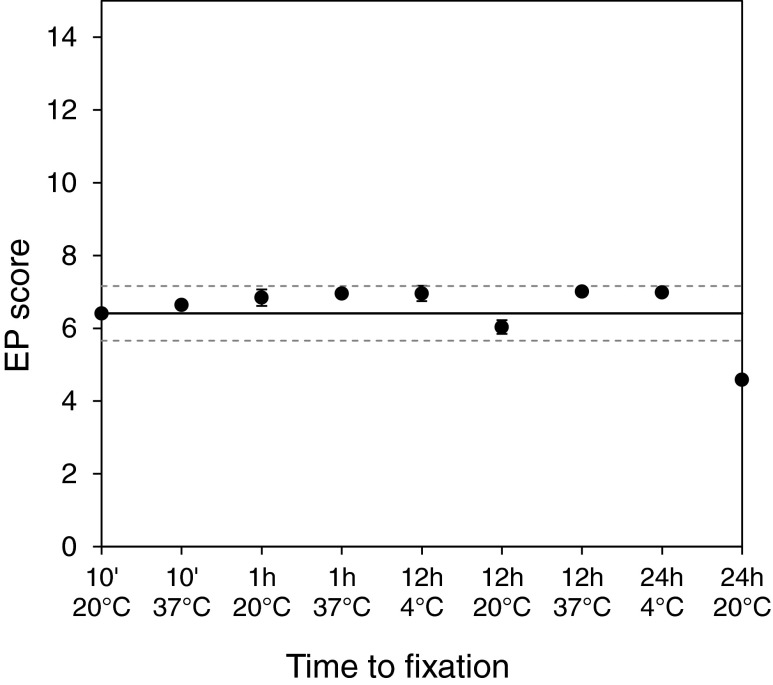
EndoPredict test results achieved after different storage times and temperatures before fixation. The *solid line* represents the reference score which was derived from the mean EP score of the tissue sections that have been stored for 10 min at 20 °C before fixation. *Dotted lines* represent the reference score ±3× its standard deviation as determined by precision study [24]. Measurements were performed in duplicate (*n* = 2). Mean values are presented, with standard errors of the means indicated by *bars*

In order to study the impact of the TTF, a breast tumor specimen was cut into several pieces which were stored for up to 24 h prior to fixation at different temperatures. The gene expression levels of the reference gene RPL37A were used as surrogate marker for the mRNA yield of the individual samples. Three replicate PCR measurements were performed for each FFPE tissue section. Mean Cq values of RPL37A were calculated based on the results of the two sections per tissue piece. The mean Cq values of RPL37A varied slightly for each tumor piece, but no trend was observed that could be related to the TTF (Table 2). EP scores of all measurements were compared to the mean EP score obtained from tissue sections that had been stored for 10 min at 20 °C prior to fixation (reference conditions). EP scores of all but one sample were within the reference EP score interval generated at reference conditions (Fig. 2). The mean deviation of these samples was 0.37 EP score units (range −0.50 to 0.70). Only the specimen with a storage time of 24 h at 20 °C before fixation showed a remarkable decrease in the EP score. The deviation compared to the reference score was 1.8 EP score units. The risk classification by EndoPredict was identical for all specimens with a TTF of up to 12 h, irrespective of the storage temperature. Even a storage time of 24 h at 4 °C before fixation did not influence the risk classification.

**Table 2:** Mean Cq values of the reference gene RPL37A of TTF and FT samples

	Mean Cq RPL37A	Standard deviation
TTF		
10 min/20 °C	22.47	0.14
10 min/37 °C	21.69	0.05
1 h/4 °C	22.30	0.33
1 h/20 °C	22.26	0.12
12 h/4 °C	22.08	0.12
12 h/20 °C	21.45	0.06
12 h/37 °C	23.61	0.05
24 h/4 °C	20.10	0.12
24 h/20 °C	21.41	0.61
FT		
1 h	20.02	0.19
6 h	28.04	0.47
20 h	20.19	0.12
2 days	20.86	0.05
5 days	22.17	0.15
10 days	20.89	0.27

### Impact of the fixation time

**Fig. 3:**
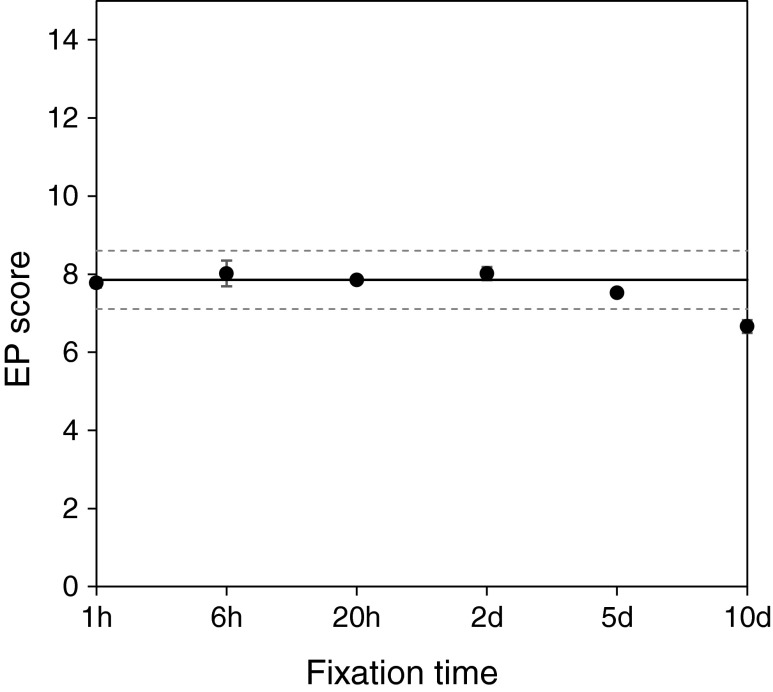
EndoPredict test results achieved after different fixation times. The *solid line* represents the reference score which was derived from the mean EP score of the tissue sections that have been fixed for 20 h in neutral buffered formalin. *Dotted lines* represent the reference score ±3× its standard deviation as determined by precision study [24]. Measurements were performed in duplicate (*n* = 2). Mean values are presented, with standard errors of the means indicated by *bars*

In order to investigate the effect of the FT on the EP score, a fresh tumor was cut into pieces which were incubated in formalin for up to 10 days. The RNA yield varied slightly for individual tissue pieces, but no trend was observed that could be related to the FT (Table 2). Mean EP score of tissue sections that had been fixed for 20 h was used as reference score (±3 standard deviations). EP scores of all specimens with FT from 1 h to 5 days were within the reference interval with a mean deviation of −0.02 EP score units from the reference EP score (range −0.40 to 0.40) (Fig. 3). A significant decrease in the EP score was only detected for tissue sections that had been fixed for 10 days. In this case, the EP score was 1.2 units lower compared to the reference score. Thus, neither a short FT of only 1 h nor longer FTs of up to 5 days affected the test results of the EndoPredict assay.

### Impact of the tumor cell content

RNA was isolated from whole tissue sections with different TCC and corresponding tumor-enriched sections with 100 % TCC. Relative expression levels of the eight genes of interest (*AZGP1*, *BIRC5*, *DHCR7*, *IL6ST*, *MGP*, *RBBP8*, *STC2*, and *UBE2C*) were compared between paired whole tissue sections and tumor-enriched specimens. Pearson correlation coefficients ranging from 0.90 to 0.98 demonstrate significant correlation of expression levels of the individual EndoPredict genes in tissue sections of different TCC (Table 3).

**Table 3:** Correlation of the relative expression values of the EndoPredict genes of interest between whole sections and manually microdissected sections

Gene name	Pearson correlation
AZGP1	0.93
BIRC5	0.94
DHCR7	0.98
IL6ST	0.90
MGP	0.93
RBBP8	0.96
STC2	0.97
UBE2C	0.95

**Fig. 4:**
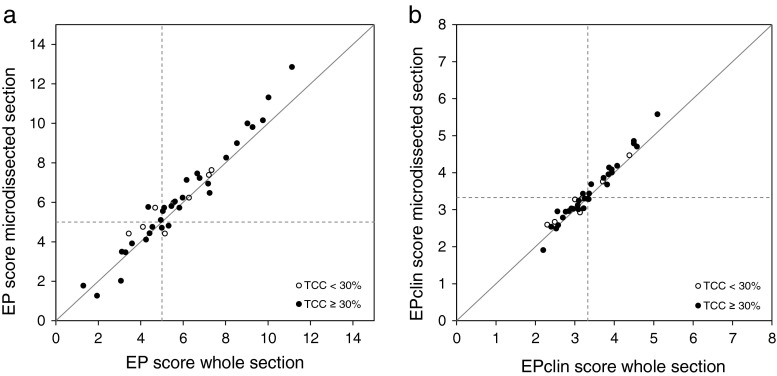
Correlation between the EP (**a**) and EPclin (**b**) scores of paired whole and tumor-enriched, manually microdissected tissue sections from ER+/HER2− breast cancer patients (*n* = 39). Pearson correlation coefficient of EP scores was 0.97 with a concordance of EndoPredict risk classification of 87 %. Pearson correlation coefficient of EPclin scores was 0.98 with a concordance of EndoPredict risk classification of 95 %. *Black filled circles* represent tissue samples with TCC ≥30 %, *open circles* tissue sections with TCC <30 %

EP scores of whole tissue sections and tumor-enriched samples showed a good correlation with a Pearson correlation coefficient of 0.97 (Fig. 4a). The mean deviation between whole and microdissected samples was −0.31 (range −1.70 to 1.10) EP score units. Similar results were obtained when considering only samples with a TCC of 30 to 100 % as recommended in the EndoPredict manual (79 % of all samples). In this case, the Pearson correlation coefficient was 0.98 with a mean deviation of −0.30 EP score units (range −1.70 to 1.10). EPclin scores were also highly correlated (*r* = 0.98) showing a mean deviation of −0.10 EPclin score units (range −0.50 to 0.30) (Fig. 4b).

The risk classification based on the EP score showed an overall agreement of 87 %. Five tumor samples that showed a disagreement between whole and manually microdissected sections had an EP score that was close to the cutoff level. Risk classification based on the EPclin score was identical for all but two tested samples (concordance of 95 %).

### Impact of the section storage time

**Fig. 5:**
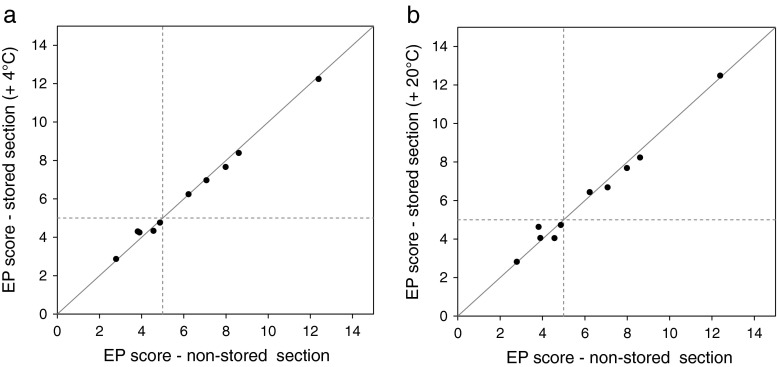
Correlation of EP scores between stored and nonstored FFPE tissue sections. EP scores have been determined at the beginning of the study, after 12 months at +4 °C (**a**) or after 12 months at +20 °C (**b**), respectively. Pearson correlation coefficients were 0.99 and >0.99 with a concordance of EndoPredict risk classification of 100 %

FFPE tissue sections of 10 breast tumors were stored for 12 months at +4 °C or for 12 months at +20 °C. Cq values of the reference gene RPL37A increased on average by 0.7 Cq values for sections stored at 4 °C and 0.9 Cq values for sections stored at +20 °C when compared to results of nonstored tissue sections indicating a decrease of RNA yield over time. Relative expression levels of individual genes, however, remained constant (Table 4). The Pearson correlation coefficient was >0.99 when comparing EP scores of samples processed without prior storage and samples stored at +4 °C (Fig. 5). It was 0.99 when doing the respective comparison between samples processed immediately and samples stored at +20 °C. Mean deviation between stored and nonstored sections was 0.02 (range −0.50 to 0.30) EP score units for storage at +4 °C and 0.04 (range −0.80 to 0.50) EP score units for storage at +20 °C. The risk classification by EndoPredict was identical for stored and nonstored sections for either condition (concordance of 100 %). Taken together, these results suggest that the discussed storage conditions have no significant impact on the EndoPredict score.

**Table 4:** Mean difference of relative expression values (delta Cq value [nonstored]-delta Cq value [stored]) of the EndoPredict genes of interest between nonstored and stored FFPE tissue sections

Gene name	Mean differences (nonstored vs 4 °C storage)	Mean differences (nonstored vs 20 °C storage)
AZGP1	0.02	0.12
BIRC5	0.06	0.33
DHCR7	0.22	0.08
IL6ST	−0.33	−0.42
MGP	−0.16	0.24
RBBP8	0.06	0.05
STC2	−0.22	−0.21
UBE2C	−0.18	−0.12

## Discussion

Invariance to tissue handling and preanalytics is a crucial requirement for RNA-based diagnostic tests. In this study, we have systematically evaluated the impact of variations of different steps in tissue handling including the TTF, the FT, and the time of FFPE tissue section storage as well as variations of the TCC on the breast cancer prognosis test EndoPredict. We could show that variations within the limits to be reasonably expected in the molecular pathology laboratory do not have a negative impact on the EndoPredict result. To our knowledge, this is the first report on the influence of TTF and FT on the results of a commercially available prognostic breast cancer gene expression test.

It is generally believed that a prolonged time prior to fixation results in a time-dependent, autolysis-induced RNA degradation that starts early upon surgical removal of the tissue [17]. Although pre-FTs should thus be kept as brief as possible, delays might occur due to transportation from the surgical room to the pathology laboratory, grossing of the specimen, or evaluation of frozen sections. It is therefore important to understand if a prolonged time period prior to fixation influences RNA yield and the EndoPredict test results. Our data indicate that a delay in fixation of up to 24 h has no effect on the RNA yield as demonstrated by mean Cq values of the reference gene RPL37A. The minor variations between the mean RPL37A Cq values are most likely caused by different sizes of the tissue pieces used. These data are in accordance with previous findings demonstrating that the RNA yield remains relatively stable in samples incubated at 4 or 25 °C for up to 24 h prior to fixation [18]. Similar results were also obtained by Godfrey and colleagues who used liver specimen from a single patient and analyzed the effect of pre-FTs on the amplification of different sized PCR amplicons of ß-actin mRNA. No differences were observed between RNA of immediately fixed specimen and samples with a pre-FT of 12 h for amplicon sizes less than 131 base pairs [19]. On the other hand, there are other studies that showed decreasing gene expression levels with a prolonged TTF [20]. Therefore, the effect of TTF on gene expression seems to be gene-specific and cannot be generalized. This has also been demonstrated by De Cecco et al. [21] who analyzed RNA isolated from subdivided breast tumors that were kept at room temperature for up to 24 h prior to freezing. Subsequent analysis was conducted with cDNA arrays containing 17.172 unique clones. The authors found that the expression of 2.88 % of genes was impacted by tissue processing times. Since some of these genes are described to play a biological role in breast cancer (e.g., *ESR1* and *ERBB2*), De Cecco et al. assumed that breast cancer signatures as well as prognostic tests could be affected by prolonged ischemic times [21]. As shown in this study, the EndoPredict test tolerates a prolonged TTF of up to 12 h. It appears that the genes included in the EndoPredict are not modulated by TTF, as are 97 % of the genes studied by De Cecco et al. The tolerance of the EndoPredict to TTF might reflect the fact that the EndoPredict assay works on highly fragmented FFPE RNA. Müller et al. found in a study with 167 FFPE breast carcinoma samples that more than 98 % of samples had RNA fragment lengths between 150 and 242 bases [22]. The PCR amplicons of the EndoPredict genes of interest are even smaller or in the same range with lengths of 68 to 157 base pairs. Another explanation for the robust expression of the EndoPredict genes could be that the final selection of the genes and the training of the algorithm were done using FFPE tissue samples from routine diagnostics [1] which presumably have had varying pre-FTs. One can therefore assume that genes, which are highly affected by prolonged pre-FTs, were already ruled out during the development of the EndoPredict test.

In a clinical setting, pre-FTs are generally kept at a minimum, and time periods of 12 h at room temperature or 24 h at +4 °C before fixation are beyond standard variations. The EP score should therefore not be affected by the TTF encountered in routine clinical practice.

Similar to the TTF, the FT can greatly vary dependent on the day time of the surgery or due to a delay in processing over weekends or holidays. Studies have shown that prolonged FTs result in poor RNA quality and decreased RNA yields. Consequently, expression level variations caused by strand breakage and protein-RNA cross-links have been observed [18, 23]. Shorter FTs had similar effects on the RNA quality. Incomplete fixation leading to tissue autolysis and inadequate dehydration might be among the reasons for the observed effects [18]. The analysis of the effect of FT on the EndoPredict test results in the present study contrasts these findings. EP scores of tissue pieces fixed for 1 h to 5 days remained stable with minor variations that were within 3-fold standard deviation of the EndoPredict score [24]. Detrimental effects were only detected after 10 days of fixation resulting in a decrease of the EP score by 1.2 units. Also, the expression levels of the reference gene RPL37A which were used as surrogate markers for the mRNA yield did not show any systematic decrease in PCR product as a function of FT. The increased mean Cq value of the sample with a FT of 6 h seems to be an outlier most likely caused by different tissue sizes since all other samples show comparable Cq values with marginal variances. Similar observations were made by Macabeo-Ong and Abrahamsen et al. who analyzed gene expression variations in relation to the FTs. Both studies showed that all or at least part of the analyzed genes showed stable expression levels after several days of fixation [20, 23]. Possible explanations for the robustness of the EndoPredict with regard to FT might also be the amplicon sizes and the development of the algorithm as explained above. Although our data is limited due to the small number of tumor samples, the results indicate that the mRNA of genes included in the EndoPredict test tolerates prolonged formalin fixation, similar to genes analyzed by Macabeo-Ong and colleagues. Moreover, our data show that a prolonged fixation of up to 5 days due to workflow variations caused by weekends or holidays does not affect EndoPredict test results.

Tumor tissue and tumor-adjacent normal breast tissue can have markedly different expression levels for individual genes. Therefore, it is reasonable that the TCC can have an impact on gene expression analysis [9, 25]. Like other breast cancer prognosis tests, EndoPredict requires a specific TCC range that must be adhered to obtain reliable results [13, 14]. For EndoPredict, this TCC range is 30 to 100 %. Our data demonstrate a good correlation of EP scores derived from whole tissue sections with TCC of 30–100 % and EP scores of consecutive, manually microdissected sections (TCC of approximately 100 %). Interestingly, even the tissue sections with a TCC of less than 30 % and the corresponding microdissected tissue samples showed comparable EP scores with a Pearson correlation coefficient of 0.91. Although the coefficient was lower compared to samples with a TCC of ≥30 %, it still indicates substantial equivalency. This indicates that the relative gene expression of the analyzed genes is even preserved in samples with a TCC below 30 %. Similar results were obtained by Tramm and colleagues who showed that the surrounding non-neoplastic tissue does not affect the quantification of *ESR1*, *PGR*, and *ERBB2* mRNA expression in breast tumor samples [26]. The test results of the PAM50-based Prosigna breast cancer prognosis test, however, are affected by adjacent nontumor tissue. This leads to changes in subtype classification or a negatively biased estimation of a patient’s risk of recurrence [27]. Accordingly, the manufacturer of Prosigna recommends to always remove the surrounding non-neoplastic breast tissue. It therefore seems that the effect of the TCC is gene-specific and needs to be evaluated specifically for each gene expression test.

Although this study analyzes a moderate number of tumor samples, the data indicates that the TCC is not an essential aspect for EndoPredict analysis. Nevertheless, to positively exclude any detrimental influence of low TCC, the requirement for EndoPredict is a minimal TCC of 30 % in the FFPE tissue section. In cases of lower TCC, a manual microdissection is recommended.

It has previously been demonstrated that reliable mRNA-based assessment of ER, PgR, and HER2 status is possible in up to 21-year-old FFPE samples [22]. Studies on FFPE tissue blocks showed an increase in fragmentation of RNA with longer storage times and increased temperature [28, 29]. Absolute expression levels as determined by qRT-PCR were affected by longer storage times, whereas relative expression levels remained constant [28]. In contrast, only limited information is available if extended storage times of FFPE tissue sections impact RNA quality. In this study, we could show that a storage time of FFPE tissue sections of up to 12 months at +4 °C or at room temperature had no influence on the EndoPredict test results. A marginal decrease of RNA yield was observed after storage at +20 °C; relative expression levels of genes of interest and EP scores, however, remained constant. Our data on EndoPredict genes is in line with the previous finding of another group showing that the storage time of FFPE tissue had only minor effects on relative gene expression values [28]. Moreover, our data demonstrate that reliable EndoPredict results can be achieved even if the tissue sections have been stored for several months after surgery.

In conclusion, our data indicates that variations in tissue handling which might occur during routine clinical procedures have negligible effects on the results of the EndoPredict test. This corroborates the statement that the EndoPredict is a robust test which can be reliably performed in an environment and workflow of a routine molecular pathological laboratory.
